# "Color Timer" mice: visualization of neuronal differentiation with fluorescent proteins

**DOI:** 10.1186/1756-6606-3-5

**Published:** 2010-02-02

**Authors:** Hiroaki Kanki, Marilia Kimie Shimabukuro, Atsushi Miyawaki, Hideyuki Okano

**Affiliations:** 1 Department of Physiology, Keio University School of Medicine, 35 Shinanomachi, Shinjuku-ku, Tokyo 160-8582, Japan; 2 Laboratory for Cell Function Dynamics, Advanced Technology Development Core, Brain Science Institute (BSI), Institute of Physical and Chemical Research (RIKEN), 2-1 Hirosawa, Wako-shi, Saitama 351-0198, Japan; 3 Current address: Departamento de Histologia e Embriologia, Instituto de Ciências Biomédicas, Universidade Federal do Rio de Janeiro (UFRJ), Rio de Janeiro, Brazil

## Abstract

The molecular mechanisms governing the differentiation of neural stem cells (NSCs) into neuronal progenitor cells and finally into neurons are gradually being revealed. The lack of convenient means for real-time determination of the stages of differentiation of individual neural cells, however, has been hindering progress in elucidating the mechanisms. In order to be able to easily identify the stages of differentiation of neural cells, we have been attempting to establish a mouse system that would allow progression of neuronal differentiation to be visualized based on transitions between fluorescence colors by using a combination of mouse genetics and the ever-expanding repertoire of fluorescent proteins. In this study we report the initial version of such a mouse system, which we call "Color Timer." We first generated transgenic (Tg; nestin/KOr Tg) mice in which production of the fluorescent protein Kusabira-Orange (KOr) is controlled by the gene regulatory elements within the 2nd intronic enhancer of the *nestin *gene, which is a good marker for NSCs, so that NSCs would emit orange fluorescence upon excitation. We then confirmed by immunohistochemical and immunocytochemical analyses that the KOr fluorescence closely reflected the presence of the Nestin protein. We also confirmed by a neurosphere formation assay that the intensity of the KOr fluorescence correlated with "stemness" of the cell and it was possible to readily identify NSCs in the two neurogenic regions, namely the dentate gyrus of the hippocampus and the subventricular zone of the lateral ventricle, in the brain of adult nestin/KOr Tg mice by the orange fluorescence they emitted. We then crossed nestin/KOr mice with doublecortin-enhanced Green Fluorescent Protein Tg mice, whose immature neurons emit green fluorescence upon excitation, and it was possible to visualize the progress of NSC-to-neuron differentiation by the transition between fluorescence colors from orange to green. This two-color initial version of the "Color Timer" mouse system will provide a powerful new tool for neurogenesis research.

## Findings

### Generation of nestin/KOr Tg mice

Identification and purification of the Green Fluorescent Protein (GFP) and cloning of its gene revolutionized the biological sciences, because labeling specific cell types, protein tracing, and real-time monitoring of gene expression in living cells, among other things, became possible for the first time [1,2]. A number of other fluorescent proteins (FPs) that possess distinct characteristics have been discovered and/or engineered since then and the ever-expanding repertoire of FPs has made it possible to label individual cells with different color of fluorescence reporters in the same animal, as, e.g., in "Brainbow" mice [3,4].

The long-term goal of our research is to establish a mouse system in which it will be possible to visualize neuronal differentiation from neural stem cells (NSCs) to neuronal progenitor cells (NPCs) and then to neurons based on transitions between the colors of the fluorescence. To achieve that goal we decided to use a combination of mouse genetics and FPs to regulate the production of different color FPs by different gene regulatory elements (GREs), each of which is specific to a different cell type, in the mouse brain.

As a first step, we attempted to establish a two-color first version of such a mouse system, which we called "Color Timer." We began by generating transgenic (Tg) mice whose NSCs, upon excitation, emit fluorescence of a color that is readily distinguishable from green, which has already been used in hundreds of reporter mouse lines. One of the most-widely-used markers for NSCs is the ~200-kDa intermediate filament protein Nestin [5]. Thorough investigation of the regulation of the *nestin *gene has revealed the importance of the second intronic enhancer as the main regulator of the NSC-specific expression of the gene [6]. A number of reporter Tg mouse lines have already been generated with this *nestin *second intronic enhancer, proving the usefulness of the element in driving expression of the gene in NSCs [7,8]. We therefore decided to use it, in combination with the *nestin *promoter, as the driver of production of the FP in our Tg mice [9].

**Figure 1:**
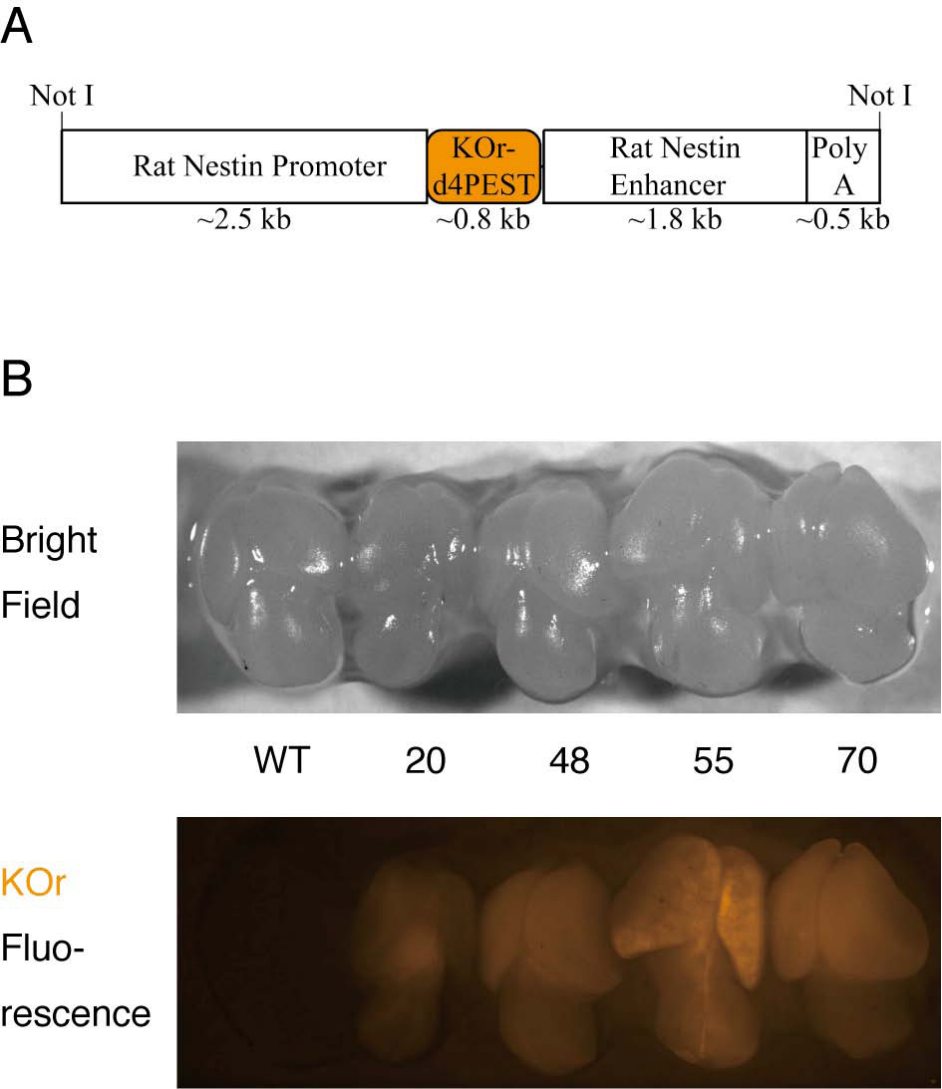
**Structure of the nestin/KOr transgene and KOr fluorescence in the brains of E14.5 transgenic mice**. **(A) **Map of the nestin/KOr transgene used to generate the transgenic mice. **(B) **Bright-field (top panel) and fluorescent (bottom panel) images of the brains of E14.5 nestin/KOr mice acquired through a stereoscopic fluorescence microscope (Leica MZ10 F) with AxioVision (Zeiss) software.

We selected the fluorescent protein Kusabira-Orange (KOr) as the reporter because of its spectral separation from GFP (Additional file 1) and red fluorescent proteins (RFPs), both of which are already being widely used in fluorescent reporter mice [10]. The "d4" version of the PEST sequence was added to the C-terminus of the KOr protein for higher temporal resolution to construct the nestin/KOr transgene with which the transgenic mice were generated (Figure 1A) and 10 of the 13 founders obtained with the transgene construct transmitted the transgene to subsequent generations.

All the animal-related procedures employed in this study were conducted according to the institutional regulations.

### Orange fluorescence of KOr in embryonic nestin/KOr Tg mouse brains

The 10 founders were crossed with wild-type B6 animals to obtain F1 progeny and they were examined for the orange fluorescence of KOr. KOr fluorescence was detected in the brains of embryonic day-14.5 (E14.5) embryos of 4 (#s 20, 48, 55, and 70) of the 10 lines (Figure 1B). As can be seen in Figure 1B, the KOr signals in the cerebral cortex of line #55 showed a scattered distribution pattern that was totally different from the patterns in the other lines. Among the other three lines, the intensity of the KOr signals was highest in line #70, intermediate in line #48, and lowest in line #20. By 8 weeks after birth (P8w), the KOr signal intensity in line #48 had become similar to its intensity in line #70, whereas the signal intensity in line #20 remained weak (data not shown).

### Overlap between the orange fluorescence of KOr and Nestin protein: Immunohistochemical analysis of nestin/KOr Tg mice

**Figure 2:**
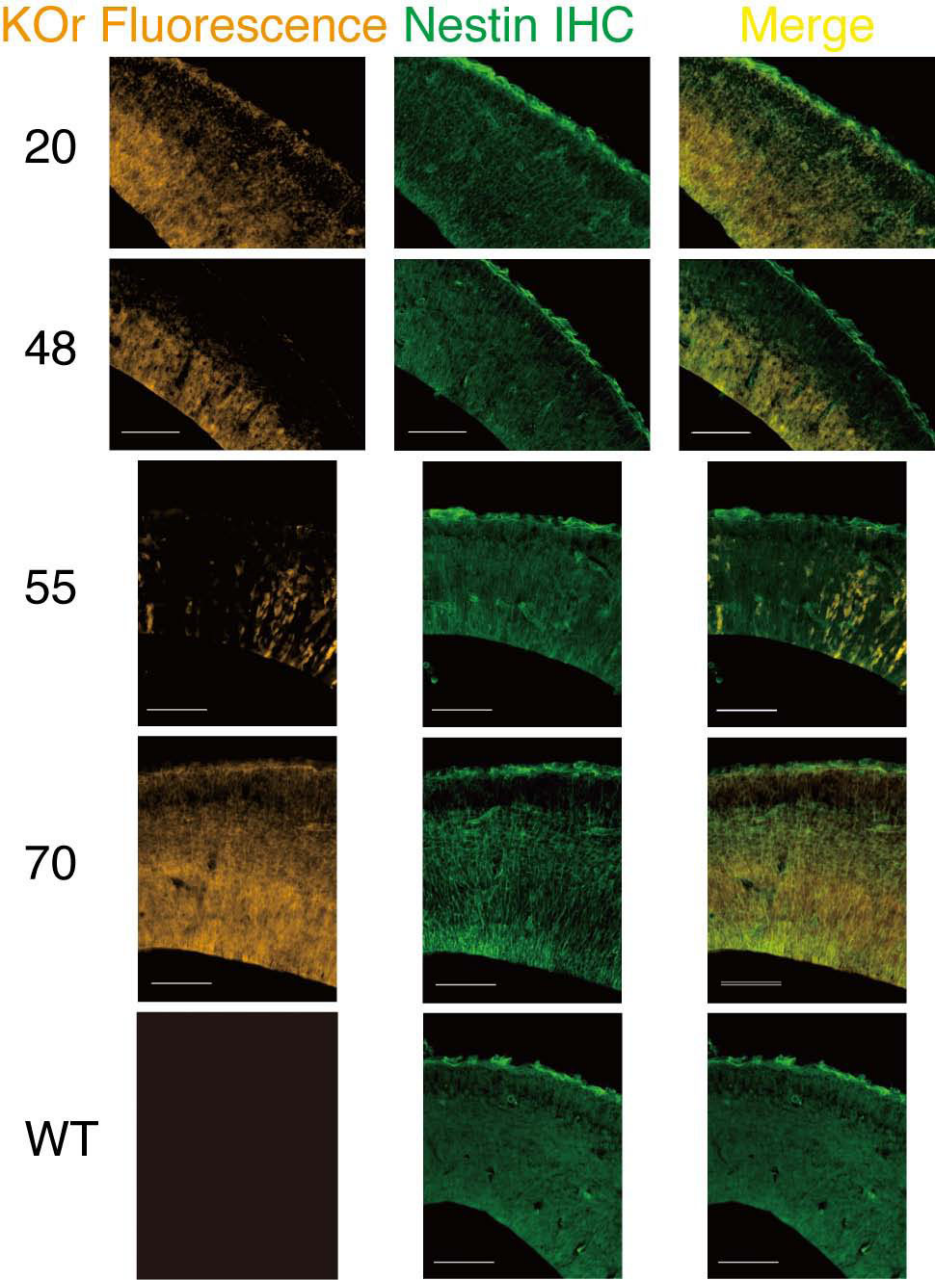
**Comparison of KOr fluorescence and Nestin protein distribution in cerebral cortices of E14.5 nestin/KOr mice**. The genotypes of E14.5 embryos were determined by direct observation of their heads under the stereoscopic fluorescence microscope described in Figure 1. KOr-positive embryos were perfused with 4% paraformaldehyde (PFA) and the brains were removed. After additional overnight fixation in 4% PFA, they were cut into 14-μm sections with a Cryostat (Leica) and then immunohistochemically (IHC) processed with a mouse anti-Nestin primary antibody (Ab; BD Biosciences Rat-401; 1:100) and FITC-conjugated donkey anti-mouse secondary Ab (Jackson ImmunoResearch; 1:250). Fluorescent micrography was performed with an ApoTome fluorescence microscope (Zeiss) and the AxioVision software (Zeiss). Scale bar: 100 μm.

In order to determine how accurately the KOr fluorescence reflected expression of the endogenous *nestin *gene and then decide which line to use in subsequent experiments, we next compared the KOr fluorescence signals and distribution of the Nestin protein, by immunohistochemical detection with an anti-Nestin antibody, in the cerebral cortices of E14.5 embryos of the 4 lines (Figure 2). KOr-positive cells exhibited radial glia-like morphology and were scattered throughout the cerebral cortex of the line #55 embryos and the intensity of the KOr signals in individual positive cells was highest in this line. Of the other three lines, line #70 showed the highest fidelity of the KOr signals, which for the most part were consistent with the signals for the immunohistochemically-detected Nestin protein. Similar results were obtained in line #20, and then in #48. As is often true of reporter mice, the overlap between the signals of the reporter KOr and the corresponding gene was not perfect even in the seemingly-best line #70. Some of the reasons for the differences are: (1) positional effects, (2) differences between the kinetics of degradation of the reporter KOr protein fused to the PEST sequence and the Nestin protein, and (3) different intracellular distributions of the two proteins. However, we judged the overlap between the signals in line #70 to be reasonably high and decided to mainly use this line for subsequent analyses.

### Correlation between KOr fluorescence intensity and levels of expression of the nestin gene: Immunocytochemical analysis

To investigate the correlation between the intensity of the orange fluorescence and the levels of expression of the *nestin *gene in individual KOr-positive cells, we performed an immunocytochemical analysis of cells prepared from the brains of E14.5 nestin/KOr Tg mouse embryos. Dissociated cells from line #70 animals were immunocytochemically processed with an anti-Nestin antibody, which was in turn recognized by an Alexa Fluor 488-conjugated secondary Ab. The processed cells were then analyzed for orange and green fluorescence with a cell sorter.

**Figure 3:**
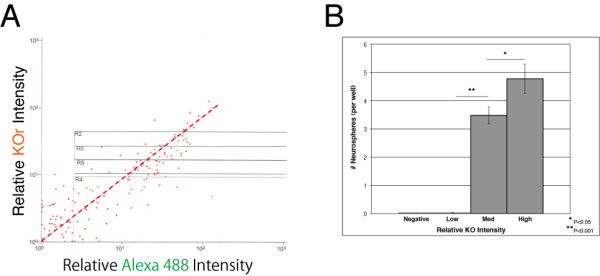
**Correlation between KOr intensity and nestin expression levels or "stemness"**. **(A) **Dissociated neural cells were prepared from the cerebral cortices of E14.5 nestin/KOr embryos and immunocytochemically labeled for Nestin by using the same anti-Nestin primary Ab described in Figure 2 and an Alexa Fluor 488-conjugated goat anti-mouse secondary Ab (Jackson ImmunoResearch; 1:250). The processed cells were then analyzed for KOr and Alexa Fluor 488 fluorescence with a cell sorter (MoFlo; Beckman Coulter). **(B) **Dissociated neural cells were prepared as in (A) and sorted into four populations according to the intensity of the KOr fluorescence. About 500 cells of each population were plated per well at a density of 2.5 cell/μl in a 96-well plate for neurosphere formation assay. After 7 days of culture in medium containing EGF (BD Biosciences) and bFGF (Roche), the numbers of spheres in each well were determined.

As shown in Figure 3A, there was a linear correlation between the intensity of the KOr fluorescence and that of the fluorescent dye Alexa Fluor 488, which reflected the amount of the Nestin protein in the cells.

These results indicated that the intensity of the KOr fluorescence closely reflected the levels of expression of the endogenous *nestin *gene in nestin/KOr mouse line #70.

### Correlation between KOr fluorescence intensity and "stemness:" Neurosphere formation assay

When cultured in a suspension containing growth factors such as epidermal growth factor (EGF) and basic fibroblast growth factor (bFGF), NSCs proliferate and form floating masses of cells called "neurospheres" and the number and size of the neurospheres have been used as a measure of "stemness" [11]. This "neurosphere formation assay" is one of the systems most widely used to evaluate the stemness of neural cells and we used it to examine the relationship between the orange fluorescence and stemness of the KOr-positive cells as described in our previous reports [7,8].

Cells were prepared from the brains of E14.5 line #70 nestin/KOr mice and after sorting them into several populations according to the intensity of the KOr fluorescence, we submitted them to a neurosphere formation assay. As shown in Figure 3B, there was a positive correlation between the intensity of the KOr fluorescence and stemness assessed by the frequency of neurosphere formation.

Based on all of these findings taken together, we concluded that NSCs can be prospectively enriched as orange fluorescent cells from nestin/KOr Tg mouse line #70, whose potency is positively correlated with the intensity of the orange fluorescence they emit.

### Orange cells in two neurogenic regions of the brains of adult nestin/KOr Tg mice

The concept of "adult neurogenesis" is now well accepted and it is widely known that new neurons are constantly generated in two neurogenic regions in the adult mammalian brain: the dentate gyrus (DG) of the hippocampus and the subventricular zone (SVZ) of the lateral ventricle (LV). Since the orange signals of KOr should be observed in these regions as new neurons are generated from NSCs, we examined the distribution of KOr-positive cells in these two regions of adult (P8w) mice.

**Figure 4:**
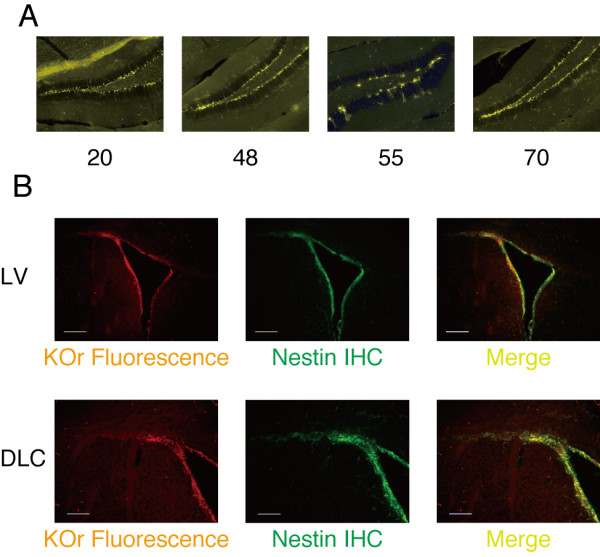
**KOr fluorescence in the two neurogenic regions in the brains of adult nestin/KOr mice**. Animals were genotyped at P2w by polymerase chain reaction (PCR) with the primer pair "hKO-Int.F" (TGAAGTACTTCATGGACGGC) and "hKO-Int.R" (TGAACTGGCACTTGTGGTTG). The resultant product was ~500 bp. **(A) **Fixed brains of P8w nestin/KOr Tg mice were cut into 40-μm sections with a Vibratome (Leica) and fluorescent micrography was performed with an ApoTome fluorescence microscope and the AxioVision software (Zeiss). **(B) **Comparison of the KOr fluorescence and the distribution of the Nestin protein around the lateral ventricle (LV; top panels) and at the dorsolateral corner (DLC; bottom panels). The slices were prepared essentially as in (A), and immunohistochemistry was performed as described in Figure 2. Scale bar: 200 μm (top row) and 100 μm (bottom row).

The orange fluorescence of KOr was easily detected in the DG of the brains of nestin/KOr Tg mice (Figure 4A). The same as in the embryonic brains, there were fewer KOr-positive cells in line #55. Among the other three lines, the intensity of the KOr signals was again highest in line #70.

In the other neurogenic area, the SVZ, the KOr fluorescence signals were distributed along the wall of the LV (Figure 4B). This pattern closely overlapped the pattern of distribution of immunohistochemically-detected Nestin protein, especially at the dorsolateral corner (bottom panels).

Taken together, these findings confirmed the usefulness of nestin/KOr Tg mouse line #70 as an NSC reporter.

### nestin/KOr - DCX-EGFP double Tg mice: the first version of the "Color Timer" mouse

We then considered which mouse line to use to fluorescence label neurons. With our future plan to separate neurons into immature and mature subpopulations in mind, we searched for a good specific marker for immature neurons.

Among the several candidates we considered, we selected Doublecortin (DCX), a ~45-kDa microtubule-associated protein already being widely used as a marker for immature neurons. The usefulness of DCX as a marker of immature neurons has been clearly demonstrated by the immunohistochemical work of the Aigner laboratory [12].

In addition to several conventional Tg mouse lines, a Tg mouse line with a bacterial artificial chromosome (BAC) backbone containing the genomic region around the mouse DCX gene was generated by the GENSAT project, whose mission is to construct an array of EGFP reporter mice by using BAC backbones with genes expressed in the nervous system [13]. This DCX-EGFP BAC Tg mouse line was used to confirm that the intensity of EGFP, which reflects the amount of the DCX protein within the cell, can be used as an indicator of neuronal maturation, which confirmed the usefulness of this mouse line as a reporter for neurons [14]. By combining this mouse line with the nestin/KOr Tg that we had generated, we hoped to be able to establish a mouse system in which the NSC-to-immature neuron differentiation could be visualized as a transition from orange to green fluorescence.

Double Tg mice were obtained by crossing nestin/KOr and DCX-EGFP animals, both of which were heterozygous, according to the Mendelian ratio as expected.

### Visualization of NSC-to-neuron differentiation by the transition from orange-to-green fluorescence in the cerebral cortex of double Tg mouse embryos

To determine whether the expected transition from orange-to-green fluorescence actually occurred during the process of NSC-to-neuron differentiation in developing mouse brains, we performed time-lapse imaging of the embryonic cerebral cortex of double Tg mice.

As expected, most of the orange cells in the double Tg animals resided on the apical side of the cortex, and the green cells were located on the cortical side. Additional file 2 shows time-lapse images of the cerebral cortex of a double Tg mouse generated by using nestin/KOr line #70. Although it is rather difficult to identify individual orange cells on the apical side of the cortex because of the ubiquitous expression of the KOr transgene in the region, an orange-to-green fluorescence transition was occasionally observed in individual cells and a typical example is indicated by an arrow in Additional file 2.

The cortex of a double Tg mouse generated by using nestin/KOr line #55 is shown in Additional file 3. The scattered distribution of KOr-positive cells made identification of yellow cells much easier than in line #70.

Taken together, these results demonstrate that the color of the fluorescence that neural cells emit upon excitation actually does change from orange to green during neuronal differentiation.

### Transition from orange-to-green fluorescence in neurospheres derived from cerebral cortices of nestin/KOr - DCX-EGFP double Tg embryos

As mentioned above, NSCs cultured in suspension in the presence of growth factors such as EGF and bFGF proliferate and form floating masses of cells called neurospheres. Since the cells undergo differentiation in parallel with self-renewal, practically all neurospheres consist of mixtures of different types of neural cells, including NSCs, neurons, and glia. To determine whether it would be possible to visualize the differentiation of NSCs into neurons in neurospheres as well, we also performed time-lapse imaging of neurospheres prepared from the cerebral cortices of E14.5 double Tg embryos with line #70 of nestin/KOr (Additional file 4).

In most of the spheres, the majority of the orange signals were found in the middle with green cells surrounding them, although the distribution of fluorescent colors was reversed (green inside and red outside) in some spheres. Yellow cells, which are presumably in the NSC-to-neuron transition phase were also observed.

### Transition from orange-to-green fluorescence along the rostral migratory stream of adult nestin/KOr - DCX-EGFP double Tg mice

**Figure 5:**
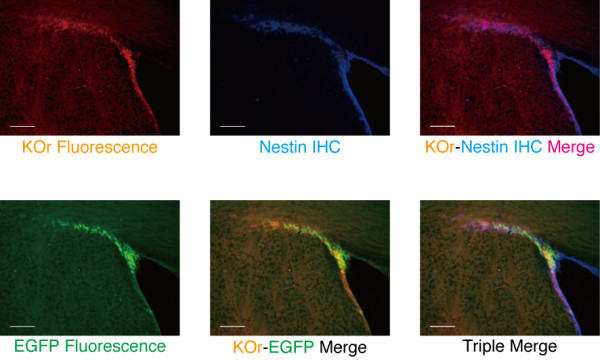
**Transition from orange to green fluorescence in RMS of adult nestin/KOr - DCX-EGFP double Tg**. Animals were genotyped at P2w by PCR with the KOr-specific primer pair (above) and a GFP-specific primer pair "GFP-Int.F3" (GCACGACTTCTTCAAGTCCGCCATGCC) and "GFP-Int.R3" (GCGGATCTTGAAGTTCACCTTGATGCC). The size of the PCR product for GFP was 265 bp. Nestin IHC was performed with the same primary Ab as in Figure 2 and an Alexa 350-conjugated anti-mouse secondary Ab (Jackson ImmunoResearch; 1:250). Scale bar: 100 μm.

Newborn neurons generated in the SVZ of the adult mouse brain migrate to the olfactory bulb and in the process they give rise to a characteristic formation called the rostral migratory stream (RMS), along which they undergo neuronal maturation. Since the RMS should provide another suitable system for investigating the relationship between neuronal differentiation and the transition between fluorescence colors in the double Tg mice, we examined the LV of an adult double Tg mouse for fluorescence (Figure 5).

KOr fluorescence was most intense along the wall of the LV, and as cells migrated and passed the dorsolateral corner, the KOr fluorescence gradually diminished and became diffuse.

The green fluorescence of EGFP, on the other hand, was relatively weak around the LV but suddenly became intense as the cells approached the dorsolateral corner, and it remained intense for some distance. Strong yellow signals, corresponding to cells in which both KOr and EGFP were present, were clustered at the dorsolateral corner.

These observations confirmed the expected orange-to-green fluorescence transition during neuronal differentiation in the adult mouse brain.

Based on all of these findings taken together, we concluded that the transition between fluorescent colors from orange to green reflects NSC-to-neuron differentiation in both the embryonic brain and the adult brain, thereby demonstrating the usefulness of these animals as an NSC-to-neuron reporter.

The pioneering work by Chalfie and colleagues clearly showed the great potential of GFP as a reporter for real-time identification of a specific cell type [15]. During the 15 years since then, thanks to the efforts of several laboratories, the FP repertoire has been expanded to the point that it now not only spans the visible spectrum, but also extends beyond it. By combining different color FPs with a number of cell-type-specific gene regulatory elements that have been identified to date, we have been trying to establish mouse systems in which neural cells can "tell what they are" by the color of the fluorescence they emit.

Based on the findings described above, we concluded that this two-color initial version of the "Color Timer" mouse system, as well as the more-advanced multi-color versions that we are currently generating, will provide a powerful new tool for neurogenesis research.

## Competing interests

The authors declare that they have no competing interests.

## Authors' contributions

HK designed the project, performed experiments, analyzed data, prepared figures, and wrote the manuscript; MKS performed experiments, analyzed data, and prepared figures; AM contributed to the original experimental design to generate the nestin/KOr Tg mice and gave various suggestions on fluorescent reporter proteins and their combinatory usages; HO provided financial supports for the experiments (except for nestin/KOr mice generation) and supervised the project. All authors read and approved the final manuscript.

## Supplementary Material

Additional file 1 **Spectral characteristics of KOr and EGFP**. The numerical data for the KOr spectra were obtained at https://ruo.mbl.co.jp/product/flprotein/ko.html/. Click here for file

Additional file 2 **Transition from orange to green fluorescence in the cerebral cortex of embryonic nestin/KOr (line #70) - DCX-EGFP double Tg mice**. Cerebral cortices of E14.5 double Tg mice with line #70 of nestin/KOr were isolated, manually cut with a knife, embedded in a collagen (Nitta Gelatin) matrix, and time-lapse imaged with an LSM5 PASCAL fluorescence microscope (Zeiss). The 543-nm HeNe laser and 488-nm Argon laser were used to excite KOr and EGFP, respectively. The cell indicated by the arrow is a representative example of the orange-to-green transition. Click here for file

Additional file 3 **Transition from orange to green fluorescence in nestin/KOr (line #55) - DCX-EGFP double Tg mice**. The cerebral cortex of an E14.5 double Tg mouse embryo with line #55 of nestin/KOr was time-lapse imaged. Click here for file

Additional file 4 **Transition from orange to green fluorescence in neurospheres derived from cerebral cortex of embryonic nestin/KOr (Line #70) - DCX-EGFP double Tg**. Cerebral cortices of E14.5 double Tg mouse embryos with line # 70 of nestin/KOr were isolated, dissociated into individual cells by mechanical pipetting, cultured for several days in a medium containing EGF and bFGF, and time-lapse imaged. Click here for file
