# Supplementary figures and images for: "Color Timer" mice: visualization of neuronal differentiation with fluorescent proteins
Source: Mol Brain. 2010 Feb 2;3:5. doi: 10.1186/1756-6606-3-5 (PMC2825511; doi:10.1186/1756-6606-3-5)

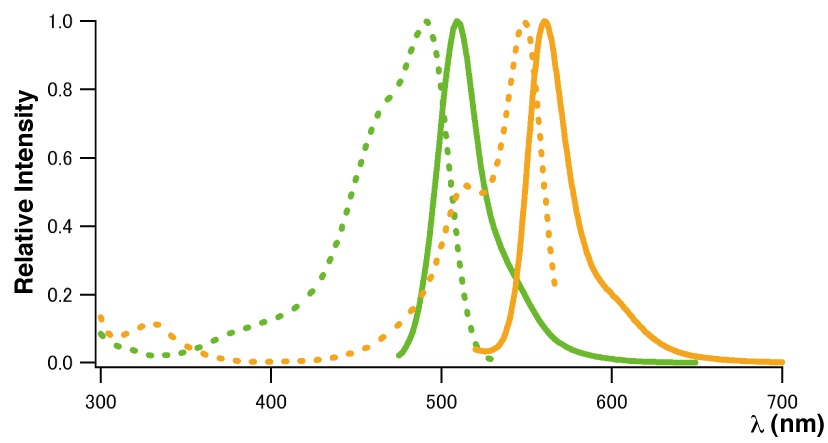

Supplement: Additional file 1 — Spectral characteristics of KOr and EGFP. The numerical data for the KOr spectra were obtained at https://ruo.mbl.co.jp/product/flprotein/ko.html/. [file 1756-6606-3-5-S1.PNG]
